# Symptoms, problems and quality of life in patients newly diagnosed with oesophageal and gastric cancer – a comparative study of treatment strategy

**DOI:** 10.1186/s12885-022-09536-x

**Published:** 2022-04-21

**Authors:** Karin Dalhammar, Jimmie Kristensson, Dan Falkenback, Birgit H. Rasmussen, Marlene Malmström

**Affiliations:** 1 Institute for Palliative Care, Lund University and Region Skåne, Lund, Sweden; 2 Department of Health Sciences, Faculty of Medicine, Lund University, Lund, Sweden; 3 Department of Surgery, Skåne University Hospital, Lund, Sweden; 4 Department of Clinical Sciences Lund, Faculty of Medicine, Lund University, Lund, Sweden

**Keywords:** Quality-of-life, Symptoms, Problems, Palliative care, Oesophageal cancer, Gastric cancer

## Abstract

**Background:**

Patients with oesophageal and gastric cancer have a low likelihood of being cured and suffer from a broad spectrum of symptoms and problems that negatively affect their quality-of-life (QOL). Although the majority (67–75%) of patients at the time of diagnosis suffer from an incurable disease, research has primarily focused on the pre- and postoperative phase among patients treated with curative intent, with little attention to symptoms and problems in the diagnostic phase, especially in those who cannot be offered a cure.

**Methods:**

In this cross-sectional study 158 patients newly diagnosed with oesophageal and gastric cancer visiting the surgical outpatient department for a preplanned care visit were included consecutively during 2018–2020. The validated instruments QLQ-C30 and QLQ-OG25, developed by the European Organization for Research and Treatment of Cancer (EORTC), and selected items from the Integrated Patient Outcome Scale (IPOS) were used to assess QOL, symptoms and problems. Differences between patients with a curative and a palliative treatment strategy were analysed using *t*-test and Mann–Whitney U test. The QLQ-C30 and QLQ-OG25 scores were compared to published reference data on the general Swedish population.

**Results:**

Among all, the QOL was markedly lower, compared with general Swedish population (mean ± SD, 55.9 ± 24.7 vs 76.4 ± 22.8, *p* < 0.001). Compared to general population, the patients had significant impairment in all QOL aspects, particularly for role and emotional functioning and for symptoms such as eating-related problems, fatigue, insomnia and dyspnea. Majority of patients also reported severe anxiety among family and friends. Among patients with oesophageal cancer those with a palliative treatment strategy, compared with curative strategy, reported significantly lower QOL (mean ± SD, 50.8 ± 28.6 vs 62.0 ± 22.9 *p* = 0.030), physical (65.5 ± 22.6 vs 83.9 ± 16.5, *p* < 0.001) and role functioning (55.7 ± 36.6 vs 73.9 ± 33.3, *p* = 0.012), and a higher burden of several symptoms and problems. No significant differences between treatment groups were shown among patients with gastric cancer.

**Conclusions:**

Patients newly diagnosed with oesophageal and gastric cancer, and especially those with incurable oesophageal cancer, have a severely affected QOL and several burdensome symptoms and problems. To better address patients’ needs, it seems important to integrate a palliative approach into oesophageal and gastric cancer care.

## Introduction

It is well known that timely and efficient care and support is essential to enhance quality of life (QOL) for patients with cancer. To enable such care we need comprehensive knowledge about how problems and needs vary depending on treatment strategy and how these are perceived by the patients at the time of diagnosis.

Patients suffering from oesophageal and gastric cancer have a low expected 5-year survival rate (16–17%) and suffer from severely hampered QOL, manifested in a broad spectrum of symptoms and problems [1–4]. For the majority of these patients (67–75%), a curatively intended treatment is not possible owing to severe comorbidity and/or advanced disease. This means that they often are offered palliative chemotherapy, radiotherapy or surgery aiming to maintain QOL, mitigate symptoms and prolong survival [2, 5, 6].

Previous research has primarily focused on the pre- and post-treatment period for patients who undergo treatment with curative intent, with little attention to prognosis and/or initial treatment strategy. This despite the fact that those with the poorest prognosis have the greatest risk of receiving poor quality care [7]. This means that the main focus in previous research has been on a subgroup of patients representing only 40% of the patient population while evidence regarding symptoms and problems in the diagnostic phase, and the potential differences with regard to initial treatment strategy, is still lacking. More knowledge about symptoms, problems and QOL, early after diagnosis of oesophageal and gastric cancer, and how these differ across patients with different treatment strategies, is therefore highly relevant to facilitate timely allocation of support for all.

Oesophageal and gastric cancer are among the ten most common malignancies worldwide [8]. In Sweden there are about 1,300 new cases of oesophageal and gastric cancer annually and the diseases cause about 1,000 deaths each year [9, 10]. Regardless of treatment strategy, patients with oesophageal and gastric cancer are burdened by disease-specific and treatment- related symptoms and problems, including dysphagia, fatigue, pain, weight loss, changed bowel habits and psychological distress, which negatively impacts their QOL [3, 11, 12]. Research also indicates that these complex symptoms and problems arise already at an early stage of the disease trajectory, indicating a need for early and proactive symptom management [13–16]. Proactive symptom management is associated with improved patient QOL, increased treatment compliance, reduced hospitalizations, and use of unplanned care [17, 18]. Early identification of patients with a high level of symptomatology, and timely support, regardless of treatment strategy, are therefore crucial to optimize the patients’ wellbeing and care.

Given the poor prognosis, a palliative care approach that aims to improve QOL and decrease suffering associated with the life-threatening illness, by anticipating, preventing and treating pain and other symptoms and problems, is particularly important for patients with oesophageal and gastric cancer [19]. The American Society of Clinical Oncology (ASCO) advocates integration of a palliative care approach within 8 weeks of diagnosis [20] to ensure proactive support and optimize QOL in patients with poor prognosis. Findings from several studies indicate that a palliative care approach integrated early in the disease trajectory not only improves QOL but also enhances symptom control and reduces health care service use [21–23]. Temel et al. noted differential effects of a specific early palliative care intervention in patients with different cancer diagnoses, suggesting that effective palliative care needs to be tailored to the specific symptoms, problems and care needs of each unique patient [24]. In the early stage of a cancer disease, symptoms and problems can differ based on disease and patient-related factors, such as age, gender and cancer stage, but treatment-related aspects can also play a role [16, 25, 26]. It has, for instance, been reported that patients receiving palliative treatment have a poorer QOL, higher symptom burden and more problems related to physical, social and emotional function, in comparison with patients with a curative treatment strategy [27]. Among patients newly diagnosed with oesophageal and gastric cancer it has been shown that symptoms and problems differ depending on age and gender, but whether and to what extent there are differences with regard to the patients’ planned treatment needs to be further investigated [13–16]. Such knowledge is important to comprehensively understand patients’ care needs in the early phase of the oesophageal and gastric cancer trajectory and to enable an anticipatory care approach tailored to the patients’ needs.

The aim of this study was to describe and compare symptoms, problems and quality of life among patients newly diagnosed with oesophageal and gastric cancer with a curative or palliative treatment strategy.

## Method

### Design

This study had a cross-sectional design.

### Study population and setting

The sample comprised 158 patients with newly diagnosed oesophageal and gastric cancer. The patients were recruited at their first visit to the surgical outpatient department at a university hospital in Sweden during 2018–2020. At this stage the decision about treatment strategy is not final, meaning that included patients are still not informed about their treatment regimen. Inclusion criteria were: patients newly diagnosed with oesophageal or gastric cancer, age ≥ 18 years, cognitive ability to participate, and ability to communicate in Swedish.

### Inclusion

All patients who met the inclusion criteria were consecutively invited to participate by a nurse or a research assistant in connection with a pre-planned care visit. At inclusion all patients were given both verbal and written information about the study.

**Fig. 1:**
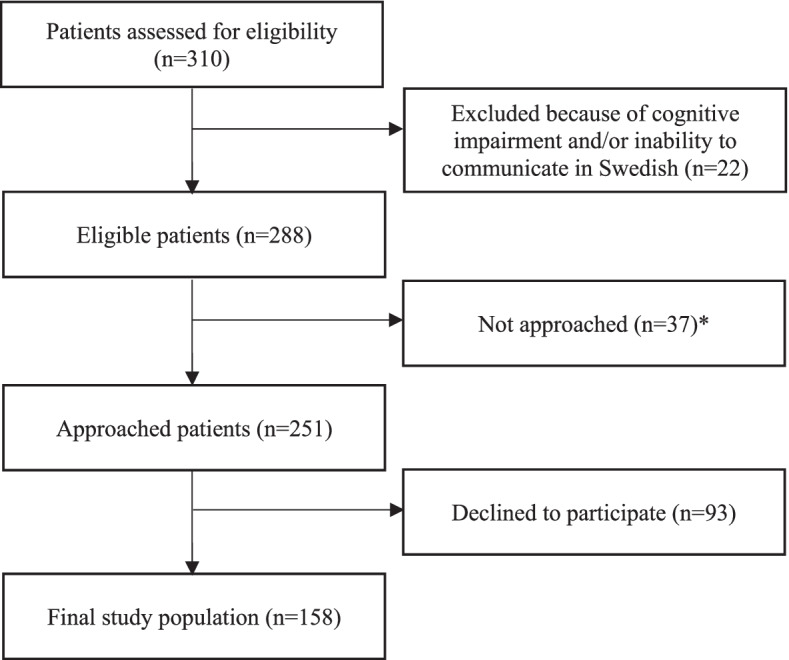
Flow diagram of the recruitment procedure. *Not approached because of administrative factors (research assistant unavailable and insufficient time for recruitment)

Patients who consented to participate were asked to complete a questionnaire at the outpatient department or at home, depending on their preference. A free-postmarked envelope was given to patients who chose to return the questionnaire by mail. Patients who did not return the questionnaire within 2 weeks were reminded by telephone up to two times by a nurse or research assistant. In total 288 patients were eligible for inclusion; 251 were approached and 158 were included in the study (Fig. 1).

### Data collection

Data were collected at inclusion through questionnaires comprising validated instruments, as well as through the patients’ medical records.

The questionnaires included the European Organization for Research and Treatment of Cancer (EORTC) validated instruments QLQ-C30 [28] and QLQ-OG25 [29], which were used to assess QOL. The cancer-specific health-related QOL questionnaire QLQ-C30 consists of 30 questions in 15 subscales: five scales focusing on function (physical, social, role, and cognitive and emotional), one scale for global QOL, three symptom scales (fatigue, pain, vomiting/nausea) and six single-item scales (insomnia, appetite loss, dyspnoea, constipation, diarrhoea, financial difficulties). The QLQ-OG25 is an oesophago-gastric-specific module comprising 25 questions in six subscales (dysphagia, eating restrictions, reflux, odynophagia, pain and anxiety). All items are scored on a 4-point Likert scale ranging from 1 (not at all) to 4 (very much), with the exception of two global QOL-related items, which use a 7-point Likert scale. Both questionnaires have demonstrated good psychometric properties [28, 29].

Data regarding clinical characteristics: tumour site, clinical M stage (M1 = the cancer has metastasized; M0 = no metastasis), histology, American Society of Anesthesiologists (ASA) score (1–6, with lower values representing better physical status) [30], and whether the initially planned treatment strategy was curative (tumour-directed treatment such as surgery/ chemotherapy/radiotherapy with a curative intent) or palliative (tumour-directed treatment or no tumour-directed therapy in non-curative patients) were collected from the medical records.

As part of the clinical routine, symptoms, problems, performance status and need for information and support are assessed by a contact nurse. The assessment is based on the Swedish Palliative Care Guide (S-PCG), which comprises single items, and the instrument Integrated Patient Outcome Scale (IPOS) [31]. The single items include questions concerning need for support and performance status according to the Eastern Cooperative Oncology Group scale (0–5, with lower values representing better function) [32]. The IPOS comprises eight items relating to physical symptoms, and psychological, emotional and spiritual, and information and support needs. All items are scored on a 5-point Likert scale ranging from “not at all” to “overwhelming/all the time” [33]. The instrument has been translated into and validated in a Swedish context [34]. Data on problems that were not covered by the QLQ-C30 and QLQ-OG25 were collected from the IPOS, using the items on anxiety among family and friends, feeling at peace, sharing feelings, information, and practical matters.

### Statistical analysis

The sample (*n* = 158) was divided into four groups according to site of primary tumour (oesophageal or gastric cancer) and the initially planned treatment strategy (curative or palliative).

Data on demographic and clinical characteristics were analysed with descriptive and analytical statistics. Differences were calculated using *t*-test for numerical data and chi-square test or Fisher’s exact test for nominal data.

The QLQ-C30 and QLQ-OG25 answers were linearly transformed to a 0–100 scale and processed using the EORTC scoring manual [35]. A high score for the global and the functional scales indicates better QOL or functioning, while a high score on the symptom scale indicates higher level of symptomatology or problems. The QLQ-C30 summary score was calculated from the mean of the 13 QLQ-C30 scales (the global QOL scale and the financial impact scale were not included). Differences in mean score were analysed using *t*-test. To ease the interpretation of the QLQ-C30 and the QLQ-OG25 results for the whole study population, a comparison to published reference data on the general Swedish population (reflecting the age and sex distribution of patients with oesophageal or gastric cancer) [25, 36], were tested with *t*-test, whenever data were available for both groups. No statistical adjustments were made. For statistically significant result, the absolute difference between mean scores were calculated. Based on previous research, a difference in mean score of ≥ 10 on the 0–100 scale of QLQ-C30, was considered clinically relevant [37]. No information on clinical relevance was presented for QLQ-OG25, as data were not available.

The median and interquartile range (IQR) were calculated for the IPOS items; differences in median were analysed using Mann–Whitney U test.

The statistical analyses were performed using version 25 of IBM SPSS Statistics (IBM Corp., Armonk, NY, USA). A *p*-value of < 0.05 was used to define statistical significance.

### Ethical approval and ethical considerations

The study was approved by the Lund Regional Ethics Review Board (REC number: 2018/03, 2018/270, 2020/03596).

## Results

### Patient characteristics

Of the included 158 patients, 113 (73.4%) were men and the mean age at diagnosis was 71.0 (SD ± 9.1) years. Most patients were married or lived in a civil partnership (113; 73.4%), were pensioners (109; 72.7%) and had children (139; 90.3%). The most common type of residence was single-family house/link house (82; 53.2%). Elementary school was the highest educational level for the majority (77; 51.3%) (Table 1).

**Table 1:** Clinical characteristics and sociodemographic data, by initial treatment strategy

Patient characteristic	Total *n* = 158	Oesophageal cancer *n* = 107 (67.7%)	Curative *n* = 65 (60.7%)	Palliative *n* = 42 (39.3%)	*p*-value	Gastric cancer *n* = 51 (32.3)	Curative *n* = 34 (66.7%)	Palliative *n* = 17 (33.3%)	*p*-value
**Age,** yrs, mean ± SD^1^	71.0 ± 9.1	70.8 ± 7.8	69.0 ± 7.4	73.4 ± 7.8	0.004	71.5 ± 11.4	69.6 ± 10.8	75.4 ± 12.0	0.086
**Weight loss,** last 6 months^2^, kg	4.8 ± 5.0	4.9 ± 4.9	4.9 ± 5.1	4.9 ± 4.7	0.980	4.4 ± 5.3	4.7 ± 6.2	3.8 ± 3.0	0.679
**BMI**^3^	25.2 ± 4.1	25.3 ± 4.3	25.3 ± 4.4	25.3 ± 4.1	0.935	25.1 ± 3.7	25.9 ± 3.6	23.2 ± 3.3	0.022
**Gender**^4^					0.232				0.771
Men, n (%)	113 (73.4)	84 (80.0)	48 (76.2)	36 (85.7)		29 (59.2)	20 (60.6)	9 (56.3)	
Women, n (%)	41 (26.6)	21 (20.0)	15 (23.8)	6 (14.3)		20 (40.8)	13 (39.4)	7 (43.8)	
**Marital status,** n (%)^5^					0.006^a^				0.515^a^
Married/civil partnership	113 (73.4)	79 (75.2)	48 (76.2)	31 (73.8)		34 (69.4)	25 (75.8)	9 (56.3)	
Divorced or separated	13 (8.4)	8 (7.6)	2 (3.2)	6 (14.3)		5 (10.2)	3 (9.1)	2 (12.5)	
Widowed	15 (9.7)	9 (8.6)	4 (6.3)	5 (11.9)		6 (12.2)	3 (9.1)	3 (18.8)	
Unmarried	13 (8.4)	9 (8.6)	9 (14.3)	–		4 (8.2)	2 (6.1)	2 (12.5)	
**Children,** n (%)^6^					0.519 ^a^				0.588 ^a^
Yes	139 (90.3)	94 (89.5)	55 (87.3)	39 (92.9)		45 (91.8)	31 (93.9)	14 (87.5)	
No	15 (9.7)	11 (10.5)	8 (12.7)	3 (7.1)		4 (8.2)	2 (6.1)	2 (12.5)	
**Type of residence,** n (%)^7^					0.026 ^a^				0.005 ^a^
Tenancy/tenants’ association	40 (26.0)	27 (25.7)	10 (15.9)	17 (40.4)		13 (26.5)	7 (21.2)	6 (37.5)	
Owner-occupied home/flat	29 (18.8)	19 (18.1)	12 (19.0)	7 (16.7)		10 (20.4)	4 (12.1)	6 (37.5)	
Single-family house/link house	82 (53.2)	57 (54.3)	39 (61.9)	18 (42.9)		25 (51.0)	22 (66.7)	3 (18.8)	
Nursing home	1 (0.6)	–	–	–		1 (2.0)	–	1 (6.3)	
Other	2 (1.3)	2 (1.9)	2 (3.2)	–		–	–	–	
**Highest education level,** n (%)^8^					0.901 ^a^				0.966 ^a^
Elementary school	77 (51.3)	53 (52.0)	31 (50.8)	22 (53.7)		24 (50.0)	15 (46.9)	9 (56.3)	
Upper secondary school, 2 years	21 (14.0)	15 (14.7)	9 (14.8)	6 (14.6)		6 (12.5)	4 (12.5)	2 (12.5)	
Upper secondary school, 3–4 years	25 (16.7)	14 (13.7)	8 (13.1)	6 (14.4)		11 (22.9)	8 (25.0)	3 (18.8)	
University < 3 years	6 (4.0)	6 (5.9)	3 (4.9)	3 (7.3)		–	–	–	
University ≥ 3 years	21 (14.0)	14 (13.7)	10 (16.4)	4 (9.8)		7 (14.6)	5 (15.6)	2 (12.5)	
**Employment status,** n (%)^9^					0.652 ^a^				1.000 ^a^
Employee	29 (19.3)	19 (18.8)	14 (22.6)	5 (12.8)		10 (20.4)	7 (21.2)	3 (18.8)	
Self-employed	9 (6.0)	7 (6.9)	4 (6.5)	3 (7.7)		2 (4.1)	1 (3.0)	1 (6.3)	
Pensioner	109 (72.7)	73 (72.3)	42 (67.7)	31 (79.5)		36 (73.5)	24 (72.7)	12 (75.0)	
Long-term sick leave	2 (1.3)	1 (1.0)	1 (1.6)	–		1 (2.0)	1 (3.0)	–	
Other	1 (0.7)	1 (1.0)	1 (1.6)	–		–	–		
**Histological type,** n (%)^10^					0.405 ^a^				
Adenocarcinoma	111 (72.5)	68 (65.4)	43 (68.3)	25 (61.0)		43 (87.8)	28 (87.5)	15 (88.2)	1.000 ^a^
Squamous cell carcinoma	32 (20.9)	32 (30.8)	19 (30.2)	13 (31.7)		–	–	–	
Other	10 (6.5)	4 (3.8)	1 (1.6)	3 (7.3)		6 (12.2)	4 (12.5)	2 (11.8)	
**M stage at diagnosis,** n (%)					< 0.001				< 0.001 ^a^
M0	105 (66.5)	68 (63.6)	57 (87.7)	11 (26.2)		37 (72.5)	33 (97.1)	4 (23.5)	
M1	53 (33.5)	39 (36.4)	8 (12.3)	31 (73.8)		14 (27.5)	1 (2.9)	13 (76.5)	
**ASA score**^11^					0.005 ^a^				1.000 ^a^
1	7 (6.8)	5 (7.4)	5 (10.0)	–		2 (5.4)	2 (6.9)	–	
2	47 (45.6)	26 (39.4)	23 (46.0)	3 (18.8)		21 (56.8)	16 (55.2)	5 (62.5)	
3	46 (44.7)	32 (48.5)	22 (44.0)	10 (62.5)		14 (37.8)	11 (37.9)	3 (37.5)	
4	3 (2.9)	3 (4.5)	–	3 (18.8)		–	–	–	
**Performance status score,** n (%)^12^					< 0.001 ^a^				0.904 ^a^
0	68 (49.6)	44 (47.3)	34 (59.6)	10 (27.8)		24 (54.5)	15 (51.7)	9 (60.0)	
1	57 (41.6)	42 (45.2)	23 (40.4)	19 (52.8)		15 (34.1)	10 (34.5)	5 (33.3)	
2	9 (6.6)	4 (4.3)	–	4 (11.1)		5 (11.4)	4 (13.8)	1 (6.7)	
3	3 (2.2)	3 (3.2)	–	3 (8.3)		–	–	–	

^a^Fisher’s exact test

*ASA* American Society of Anesthesiologists, *BMI* body mass index, *SD* standard deviation

Missing: ^1^4; ^2^76; ^3^10; ^4^4; ^5^4; ^6^4; ^7^4; ^8^8; ^9^8; ^10^5; ^11^55; ^12^21

Among all patients, 107 (67.7%) had a tumour originating in the oesophagus and 53 (33.5%) had distant metastases (M1) at the time of diagnosis. Adenocarcinoma and squamous cell carcinoma accounted for 72.5% and 20.9% of all cancers, respectively. Most patients had an ASA score of 2 (47; 45.6%) and a performance status score of 0 (68; 49.6%). The patients had a mean body mass index (BMI) of 25.2 (SD ± 4.1) and had lost 4.8 kg (SD ± 5.0) during the last 6 months prior to diagnosis (Table 1).

### Quality of life, symptoms and problems among patients newly diagnosed with oesophageal and gastric cancer

The patients had a mean score of 55.9 (SD ± 24.7) for self-reported global health status/QOL. On the functional scales, the patients reported lowest score for role functioning (mean ± SD, 65.4 ± 36.1), followed by emotional functioning (69.3 ± 24.8) and social functioning (74.9 ± 28.5). Within the symptom scales, the patients reported the highest score for appetite loss (42.1 ± 40.7), fatigue (40.2 ± 27.9) and insomnia (35.5 ± 33.3). The most prominent diagnosis-specific symptoms reported by all participants were anxiety (mean ± SD, 65.1 ± 28.4), eating restrictions (45.8 ± 35.1) and odynophagia (35.6 ± 33.7) (Table 2).

**Table 2:** Quality-of-life among patients newly diagnosed with oesophageal and gastric cancer compared with general Swedish population

Questionnaire item	Study population *n* = 158	Reference population^a^ *n* = 4910	*p*-value	Absolute difference between means
**QLQ-C30,** mean ± SD				
Global health status^1^	55.9 ± 24.7	76.4 ± 22.8	** < 0.001**	**20.5**^**c**^
Summary score^2^	73.1 ± 18.6	*No reference data available*	-	-
Function^*^				
Physical function^3^	76.9 ± 21.2	88.0 ± 18.3	** < 0.001**	**11.1**^**c**^
Role function^4^	65.4 ± 36.1	82.2 ± 23.9	** < 0.001**	**16.8**^**c**^
Emotional function^5^	69.3 ± 24.8	85.8 ± 18.7	** < 0.001**	**16.5**^**c**^
Cognitive function^6^	84.6 ± 21.4	88.1 ± 16.9	**0.011**	3.5
Social function^7^	74.9 ± 28.5	91.2 ± 19.0	** < 0.001**	**16.3**^**c**^
Symptom^**^				
Fatigue^8^	40.2 ± 27.9	19.1 ± 21.7	** < 0.001**	**21.1**^**c**^
Nausea and vomiting^9^	20.2 ± 26.9	2.6 ± 9.3	** < 0.001**	**17.6**^**c**^
Pain^10^	27.8 ± 31.7	18.9 ± 25.7	** < 0.001**	8.9
Dyspnoea^11^	28.8 ± 31.0	16.3 ± 24.3	** < 0.001**	**12.5**^**c**^
Insomnia^12^	35.5 ± 33.3	17.5 ± 25.9	** < 0.001**	**18.0**^**c**^
Appetite loss^13^	42.1 ± 40.7	3.3 ± 12.8	** < 0.001**	**38.8**^**c**^
Constipation^14^	24.1 ± 31.8	5.4 ± 6.1	** < 0.001**	**18.7**^**c**^
Diarrhoea^15^	11.0 ± 20.2	5.6 ± 15.9	** < 0.001**	5.4
Financial difficulties^16^	7.5 ± 19.8	4.4 ± 16.2	**0.019**	3.1
QLQ-OG25^**^, mean ± SD		**Reference population**^**b**^		
Dysphagia^17^	33.3 ± 33.2	0.8 ± 5.5	** < 0.001**	32.5^**d**^
Eating restrictions^18^	45.8 ± 35.1	2.9 ± 9.9	** < 0.001**	42.9^**d**^
Reflux^19^	17.3 ± 26.0	6.7 ± 15.4	** < 0.001**	10.6^**d**^
Odynophagia^20^	35.6 ± 33.7	1.5 ± 8.23	** < 0.001**	34.1^**d**^
Pain and discomfort^21^	27.9 ± 33.9	7.6 ± 16.9	** < 0.001**	20.3^**d**^
Anxiety^22^	65.1 ± 28.4	*No reference data available*	-	-
Eating with others^23^	30.2 ± 39.1	1.3 ± 8.9	** < 0.001**	28.9^**d**^
Dry mouth^24^	26.7 ± 32.7	11.5 ± 23.0	** < 0.001**	15.2^**d**^
Sense of taste^25^	15.6 ± 27.1	2.6 ± 12.5	** < 0.001**	13.0^**d**^
Body image^26^	24.5 ± 33.2	*No reference data available*	-	-
Saliva^27^	14.0 ± 26.3	1.3 ± 9.2	** < 0.001**	12.7^**d**^
Choking^28^	20.7 ± 29.8	3.7 ± 13.1	** < 0.001**	17.0^**d**^
Cough^29^	24.0 ± 27.8	13.7 ± 23.6	** < 0.001**	10.3^**d**^
Speech^30^	8.2 ± 20.5	2.2 ± 11.0	** < 0.001**	6.0^**d**^
Weight loss^31^	28.4 ± 34.9	1.8 ± 10.5	** < 0.001**	26.6^**d**^
Hair loss^32^	11.7 ± 27.1	*No reference data available*	-	-

^*^Score range 0–100. A high score represents a higher level of quality of life (QOL) or functioning. **Score range 0–100. A high score represents a higher level of symptoms

Missing: ^1^6; ^2^20; ^3^9; ^4^10; ^5^6; ^6^5; ^7^6; ^8^9; ^9^8; ^10^4; ^11^10; ^12^7; ^13^9; ^14^7; ^15^10; ^16^12; ^17^8; ^18^9; ^19^8; ^20^11; ^21^12; ^22^7; ^23^10; ^24^12; ^25^11; ^26^11; ^27^8; ^28^7; ^29^11; ^30^11; ^31^10; ^32^138

^a^QLQ-C30 mean scores of Swedish population (reflecting the age and sex distribution of patients with oesophageal or gastric cancer) used as a reference group [25]

^b^QLQ-OG25 mean scores of Swedish population (reflecting the age and sex distribution of patients with oesophageal or gastric cancer) used as a reference group [36]

^c^Clinically relevant; mean scores differ by ≥ 10 [37]

^**d**^No data on clinical relevance is available

Compared to the general Swedish population, the patients reported significantly worse mean scores for all QOL aspects; global QOL, functional performance, and general and diagnose-specific symptoms. Clinically relevant differences were shown for global QOL, all aspects of functioning, except for cognitive functioning, and for several symptoms related to eating, fatigue, insomnia and dyspnea (Table 2).

Patients reported severe anxiety among their family and friends (median 3.0; first to second quartile (Q1–Q2) 2.0–3.0) and that their own feeling of being at peace was slightly affected (1.0; 1.0–2.0). They also reported that they could always share feelings with family and friends (0.0; 0.0–2.0), that they had received as much information as they wanted (0.0; 0.0–2.0) and that their practical matters had been addressed (0.0; 0.0–1.0) (Table 3).

**Table 3:** Median values for included IPOS items among patients newly diagnosed with oesophageal and gastric cancer

**IPOS item**^a^**,** median (Q1–Q3)	**Total *n* = 158**
Anxiety among family^1^ (Have any of your family or friends been anxious or worried about you?)	3.0 (2.0–3.0)
Feeling at peace^2^ (Have you felt at peace?)	1.0 (1.0–2.0)
Sharing feelings^3^ (Have you been able to share how you are feeling with your family and friends as much as you have wanted?)	0.0 (0.0–2.0)
Information^4^ (Have you received as much information as you wanted?)	0.0 (0.0–2.0)
Practical matters^5^ (Have any practical problems resulting from your illness been addressed (such as financial or practical matters)?)	0.0 (0.0–1.0)

^a^Range 0–4. A high score represents a higher level of problems, *Q1* first quartile, *Q3* third quartile. Missing: ^1^17; ^2^17; ^3^19; ^4^23; ^5^23

### Quality of life, symptoms and problems, by treatment strategy in patients with oesophageal cancer

Among patients with oesophageal cancer, patients with a palliative treatment strategy reported a significantly lower global QOL and a lower global health summary score, in comparison with patients planned for a curative treatment strategy (mean ± SD, 50.8 ± 28.6 vs 62.0 ± 22.9 and 69.0 ± 19.9 vs 77.0 ± 17.4, respectively). Physical and role functioning were also lower among patients planned to receive palliative treatment compared with patients planned for curative treatment (65.5 ± 22.6 vs 83.9 ± 16.5 and 55.7 ± 36.6 vs 73.9 ± 33.3, respectively), while no significant differences between the groups were shown for emotional, cognitive and social functioning (Table 4).

**Table 4:** Mean values for quality of life among patients with oesophageal cancer, by treatment strategy

Questionnaire item	Oesophageal cancer				
	Total *n* = 107	Palliative *n* = 42 (39.3%)	Curative *n* = 65 (60.7%)	*p*-value	**Absolute difference between means**
**QLQ-C30,** mean ± SD					
Global health status^1^	57.5 ± 25.8	50.8 ± 28.6	62.0 ± 22.9	**0.030**	**11.2**^a^
Summary score^2^	74.0 ± 18.7	69.0 ± 19.9	77.0 ± 17.4	**0.048**	**8**
**Function**^’^					
Physical function^3^	76.8 ± 21.0	65.5 ± 22.6	83.9 ± 16.5	** < 0.001**	**18.4**^a^
Role function^4^	67.0 ± 35.5	55.7 ± 36.6	73.9 ± 33.3	**0.012**	**18.2**^a^
Emotional function^5^	69.3 ± 24.8	68.5 ± 23.5	69.8 ± 25.8	0.786	
Cognitive function^6^	86.5 ± 20.3	84.9 ± 20.1	87.6 ± 20.5	0.505	
Social function^7^	74.8 ± 29.5	74.2 ± 27.6	75.3 ± 30.9	0.858	
**Symptom**^’’^					
Fatigue^8^	38.3 ± 28.2	47.1 ± 27.3	32.5 ± 27.5	**0.010**	**14.6**^a^
Nausea and vomiting^9^	19.4 ± 25.8	24.2 ± 27.2	16.4 ± 24.6	0.138	
Pain^10^	27.6 ± 33.1	39.3 ± 37.1	19.8 ± 27.8	**0.005**	**19.5**^a^
Dyspnoea^11^	28.7 ± 31.4	39.5 ± 33.7	22.0 ± 28.3	**0.006**	**17.5**^a^
Insomnia^12^	36.2 ± 34.8	41.3 ± 34.4	32.8 ± 34.9	0.225	
Appetite loss^13^	40.5 ± 41.1	48.0 ± 44.1	35.5 ± 38.4	0.146	
Constipation^14^	22.3 ± 31.1	20.6 ± 29.4	23.5 ± 32.4	0.648	
Diarrhoea^15^	9.7 ± 17.9	12.2 ± 19.4	8.1 ± 16.7	0.252	
Financial difficulties^16^	7.7 ± 18.9	7.0 ± 17.6	8.2 ± 19.9	0.765	
**QLQ-OG25**^’’^**,** mean ± SD					
Dysphagia^17^	39.2 ± 31.9	42.3 ± 33.3	37.0 ± 31.0	0.413	
Eating restrictions^18^	50.2 ± 35.0	54.5 ± 37.7	47.3 ± 33.0	0.313	
Reflux^19^	15.9 ± 25.1	21.0 ± 30.4	12.3 ± 20.2	0.107	
Odynophagia^20^	43.3 ± 34.3	45.8 ± 38.1	41.7 ± 31.8	0.555	
Pain and discomfort^21^	27.6 ± 36.0	30.0 ± 35.2	26.0 ± 36.7	0.589	
Anxiety^22^	64.7 ± 28.7	62.3 ± 27.3	66.4 ± 29.7	0.480	
Difficulties eating with others^23^	36.6 ± 41.2	38.3 ± 43.1	35.5 ± 40.3	0.730	
Dry mouth^24^	27.3 ± 32.4	33.3 ± 33.8	23.5 ± 31.2	0.142	
Sense of taste^25^	17.7 ± 27.8	23.3 ± 33.9	13.9 ± 22.4	0.126	
Body image^26^	26.3 ± 33.8	36.8 ± 34.0	19.4 ± 32.1	**0.012**	**17.4**^**b**^
Saliva^27^	17.5 ± 29.8	23.0 ± 34.9	13.7 ± 25.4	0.141	
Choking^28^	26.9 ± 32.2	31.0 ± 36.4	24.2 ± 29.1	0.296	
Cough^29^	26.7 ± 28.4	31.7 ± 32.9	23.3 ± 24.8	0.152	
Speech^30^	9.6 ± 22.8	18.8 ± 31.3	3.8 ± 12.2	**0.006**	**15**^**b**^
Weight loss^31^	32.3 ± 36.3	33.3 ± 37.7	31.7 ± 36.4	0.829	
Hair loss^32^	15.6 ± 30.5	41.7 ± 50.0	6.1 ± 13.5	0.250	

^*^Score range 0–100. A high score represents a higher level of quality of life (QOL) or functioning. **Score range 0–100. A high score represents a higher level of symptoms

Missing: ^1^3; ^2^14; ^3^6; ^4^7; ^5^3; ^6^3; ^7^3; ^8^6; ^9^5; ^10^2; ^11^7; ^12^3; ^13^5; ^14^4; ^15^4; ^16^8; ^17^5; ^18^5; ^19^4; ^20^7; ^21^8; ^22^4; ^23^6; ^24^8; ^25^7; ^26^8; ^27^4; ^28^3; ^29^7; ^30^6; ^31^6; ^32^92

^a^Clinically relevant; mean scores differ by ≥ 10 [37]

^**b**^No data on clinical relevance is available

In comparison with patients planned for curative treatment, patients planned for palliative treatment reported significantly higher intensity of fatigue (mean ± SD, 47.1 ± 27.3 vs 32.5 ± 27.5), pain (39.3 ± 37.1 vs 19.8 ± 27.8) and dyspnea (39.5 ± 33.7 vs 22.0 ± 28.3). The mean scores in other symptom scales did not differ with statistical significance between treatment groups (Table 4).

Clinically relevant differences were shown for global QOL, physical and role functioning and fatigue, pain and dyspnea (Table 4).

Patients planned for palliative treatment reported significantly worse body image and speech in comparison with patients planned for curative treatment (mean ± SD, 36.8 ± 34.0 vs 19.4 ± 32.1 and 18.8 ± 31.3 vs 3.8 ± 12.2, respectively). No significant differences between the groups were shown for the other disease-specific symptoms (Table 4).

Among patients with oesophageal cancer, no significant differences in anxiety among family and friends, own feeling at peace, sharing feelings, and information and practical matters were found between patients with a palliative and patients with a curative treatment strategy (Table 5).

**Table 5:** Median values for included IPOS items in patients with oesophageal cancer, by treatment strategy

Questionnaire item	Oesophageal cancer			
	Total *n* = 107	Palliative *n* = 42 (39.3%)	Curative *n* = 65 (60.7%)	*p*-value
**IPOS item**^a^**,** median (Q1–Q3)				
Anxiety among family^1^ (Have any of your family or friends been anxious or worried about you?)	3.0 (2.0–3.25)	3.0 (2.0–3.0)	3.0 (2.0–4.0)	0.724
Feeling at peace^2^ (Have you felt at peace?)	1.0 (1.0–2.0)	1.5 (0.0–2.0)	1.0 (1.0–2.0)	0.826
Sharing feelings^3^ (Have you been able to share how you are feeling with your family and friends as much as you have wanted?)	1.0 (0.0–2.0)	1.0 (0.0–2.0)	0.0 (0.0–2.0)	0.610
Information^4^ (Have you had as much information as you wanted?)	0.0 (0.0–2.0)	0.0 (0.0–1.5)	0.0 (0.0–2.0)	0.240
Practical matters^5^ (Have any practical problems resulting from your illness been addressed (such as financial or practical matters)?)	0.0 (0.0–2.0)	0.0 (0.0–2.0)	0.0 (0.0–1.5)	0.877

^a^Range 0–4. A high score represents a higher level of problems, *Q1* first quartile, *Q3* third quartile

Missing: ^1^9; ^2^10; ^3^12; ^4^15; ^5^14

### Quality of life, symptoms and problems, by treatment strategy in patients with gastric cancer

Among patients with gastric cancer, there were no significant differences in QOL, functioning and symptoms between the initial treatment strategies (Table 6).

**Table 6:** Mean values for quality of life among patients with gastric cancer, by treatment strategy

Questionnaire item	Gastric cancer			
	Total *n* = 51	Palliative *n* = 17 (33.3%)	Curative *n* = 34 (66.7%)	*p*-value
**QLQ-C30,** mean ± SD				
Global health status^1^	52.6 ± 22.0	52.8 ± 22.9	52.5 ± 21.9	0.971
Summary score^2^	71.3 ± 18.5	71.0 ± 22.5	71.4 ± 16.6	0.942
**Function**^’^				
Physical function^3^	77.3 ± 21.9	75.6 ± 24.7	78.1 ± 20.8	0.714
Role function^4^	62.2 ± 37.5	65.6 ± 36.2	60.4 ± 38.5	0.655
Emotional function^5^	69.4 ± 25.2	73.0 ± 27.4	67.8 ± 24.4	0.513
Cognitive function^6^	80.6 ± 23.4	79.2 ± 28.9	81.3 ± 20.7	0.767
Social function^7^	75.0 ± 26.4	73.3 ± 32.0	75.8 ± 24.0	0.772
**Symptom**^’’^				
Fatigue^8^	44.2 ± 27.1	46.5 ± 30.4	43.1 ± 25.7	0.680
Nausea and vomiting^9^	21.9 ± 29.2	25.0 ± 29.8	20.3 ± 29.2	0.605
Pain^10^	28.2 ± 28.9	29.2 ± 33.1	27.8 ± 27.2	0.877
Dyspnoea^11^	29.2 ± 30.5	22.9 ± 31.5	32.3 ± 29.9	0.320
Insomnia^12^	34.0 ± 29.9	27.1 ± 27.8	37.6 ± 30.7	0.256
Appetite loss^13^	45.4 ± 40.2	50.0 ± 40.4	43.0 ± 40.5	0.578
Constipation^14^	27.8 ± 33.2	39.6 ± 37.0	21.9 ± 30.1	0.082
Diarrhoea^15^	13.6 ± 24.5	12.5 ± 24.0	14.1 ± 25.0	0.828
Financial difficulties^16^	7.1 ± 21.9	2.2 ± 8.6	9.4 ± 25.7	0.165
**QLQ-OG25**^’’^**,** mean ± SD				
Dysphagia^17^	20.6 ± 32.7	20.7 ± 27.5	20.5 ± 35.1	0.984
Eating restrictions^18^	36.3 ± 33.7	39.4 ± 34.1	34.8 ± 33.9	0.665
Reflux^19^	20.6 ± 27.8	20.0 ± 22.9	20.8 ± 30.2	0.925
Odynophagia^20^	19.1 ± 25.5	17.8 ± 24.0	19.8 ± 26.6	0.804
Pain and discomfort^21^	28.4 ± 29.5	37.8 ± 30.5	24.0 ± 28.4	0.136
Anxiety^22^	66.0 ± 27.9	63.3 ± 31.0	67.2 ± 26.8	0.665
Difficulties eating with others^23^	16.3 ± 30.2	11.1 ± 24.1	18.8 ± 32.7	0.425
Dry mouth^24^	25.5 ± 33.5	31.1 ± 32.0	22.9 ± 34.3	0.440
Sense of taste^25^	11.3 ± 27.2	15.6 ± 27.8	9.4 ± 27.1	0.473
Body image^26^	20.8 ± 32.0	15.6 ± 24.8	23.2 ± 34.8	0.447
Saliva^27^	6.4 ± 13.3	8.9 ± 15.3	5.2 ± 12.3	0.381
Choking^28^	7.1 ± 16.9	13.3 ± 24.6	4.2 ± 11.2	0.186
Cough^29^	18.4 ± 25.8	15.6 ± 24.8	19.8 ± 26.6	0.606
Speech^30^	5.1 ± 14.0	2.2 ± 8.6	6.5 ± 15.9	0.249
Weight loss^31^	19.9 ± 30.0	28.9 ± 33.0	15.6 ± 28.1	0.160
Hair loss^32^	0.0 ± 0.0	0.0 ± 0.0	0.0 ± 0.0	–

^*^Score range 0–100. A high score represents a higher level of quality of life (QOL) or functioning. **Score range 0–100. A high score represents a higher level of symptoms

Missing: ^1^3; ^2^6; ^3^3; ^4^3; ^5^3; ^6^2; ^7^3; ^8^3; ^9^3; ^10^2; ^11^3; ^12^4; ^13^4; ^14^3; ^15^2; ^16^4; ^17^3; ^18^4; ^19^4; ^20^4; ^21^4; ^22^3; ^23^4; ^24^4; ^25^4; ^26^3; ^27^4; ^28^4; ^29^4; ^30^5; ^31^4; ^32^46

Regarding diagnosis-specific symptoms, there were no significant differences between the treatment groups (Table 6).

Among patients with gastric cancer, no significant differences in anxiety among family and friends, own feeling at peace, sharing feelings, and information and practical matters were found between those with a palliative and those with a curative treatment (Table 7).

**Table 7:** Median values for included IPOS items in patients with gastric cancer, by treatment strategy

Questionnaire item	Gastric cancer			
	Total *n* = 51	Palliative *n* = 17 (33.3%)	Curative *n* = 34 (66.7%)	*p*-value
**IPOS item**^a^, median (Q1–Q3)				
Anxiety among family^1^ (Have any of your family or friends been anxious or worried about you?)	3.0 (2.0–3.0)	2.0 (2.0–3.0)	3.0 (2.0–3.25)	0.395
Feeling at peace^2^ (Have you felt at peace?)	1.0 (0.25–2.0)	2.0 (1.0–2.0)	1.0 (0.0–2.0)	0.319
Sharing feelings^3^ (Have you been able to share how you are feeling with your family and friends as much as you have wanted?)	0.0 (0.0–1.0)	0.5 (0.0–1.0)	0.0 (0.0–1.0)	0.611
Information^4^ (Have you had as much information as you wanted?)	0.0 (0.0–1.0)	1.0 (0.0–2.5)	0.0 (0.0–1.0)	0.380
Practical matters^5^ (Have any practical problems resulting from your illness been addressed (such as financial or practical matters)?)	0.0 (0.0–1.0)	0.0 (0.0–1.0)	0.0 (0.0–1.0)	0.893

^a^Range 0–4. A high score represents a higher level of problems. *Q1* first quartile, *Q3* third quartile

Missing: ^1^8; ^2^7; ^3^7; ^4^8; ^5^9

## Discussion

This is, to the best of our knowledge, the first study to describe symptoms, problems and QOL among patients newly diagnosed with oesophageal and gastric cancer not yet having a final treatment decision and to compare differences between those planned for curative and those planned for palliative treatment. While previous research has demonstrated a long and short-term deterioration in QOL during the course of treatment [1, 38, 39], this study shows that patients with oesophageal and gastric cancer present with a clinically relevant lower QOL and functioning and higher burden from several symptoms in comparison to the general Swedish population.

Among all, the mean global health status/QOL score was 55.9. Patients scored lowest on role and emotional functioning, while anxiety, fatigue and problems related to eating were the most predominant concerns. Although differences in sample selection and study design make comparisons with other studies difficult, these findings are in line with several previous studies [14, 16, 26, 40]. Tomaszewski et al. showed that role functioning was most impaired and that anxiety, fatigue and problems related to eating were the most common concerns in the pre-treatment phase among patients with oesophageal and gastric cancer [16]. Also, EORTC reference data revealed a mean global health status/QOL of 55.6 (SD ± 24.1) and 53.1 (± 26.5) for patients with oesophageal and gastric cancer, respectively, and that patients scored lowest on role functioning and highest on fatigue and problems related to eating [40]. Compared to the general population in Sweden, the newly diagnosed patients QOL are impaired in several ways [25, 36]. For example, their global QOL is more than 20 points and role, emotional and social functioning are about 16 points, below the mean value for the general Swedish population, differences which are considered clinically significant for the QLQ-C30 scale [25, 37]. This large discrepancy in QOL indicates that patients with oesophageal and gastric cancer are severely affected by their illness already at the time of presentation. The patients’ hampered QOL and low chance of cure suggest that a proactive palliative approach, focusing on symptom management and QOL optimization, may be beneficial already from diagnosis of oesophageal and gastric cancer. The patients’ broad spectrum of concerns, ranging from anxiety to problems with eating, also suggest that interdisciplinary support may be important to proactively address the physical and psychological needs of the patients. In the aftermath of an oesophageal or gastric cancer diagnosis, it may be especially important to provide psychological support, as anxiety can lead to fatigue and depression over time. Involvement of dieticians could prevent long-term consequences of problems related to eating [41–43]. However, the current health care service often focuses on diagnostics and medical treatment, rather than embracing a holistic approach to patient care, even though there is strong evidence that early palliative care provides benefits for patients in terms of reduced symptom burden and increased QOL [44, 45]. The present study suggests that a proactive palliative approach needs to be integrated in regular treatment regimens.

There were few differences between patients with regard to the initial treatment strategy, indicating that patients are in need of extended support, irrespective of whether they present with a curable or an incurable disease. This is consistent with ASCO guidelines, recommending access to palliative care competence and knowledge for all seriously ill patients, regardless of disease stage and treatment intent [20]. Among patients with oesophageal cancer, those with palliative treatment had a clinically relevant and statistically significant lower QOL and physical and role functioning, and a higher burden of pain, dyspnoea and fatigue, in comparison with patients receiving curative treatment. The result is similar to findings from a study by Tomaszewski et al., which showed that patients with palliative treatment had a lower level of physical functioning and higher burden of fatigue in the early stage of disease, in comparison with patients receiving curative treatment [16]. The lower physical functioning and the higher burden of fatigue, dyspnoea and pain among patients with a palliative treatment strategy could be related to their higher age, more advanced cancer stage and their lower general health status. Several studies have also shown that pain and fatigue are especially common in patients with advanced cancer [46, 47], and research has previously shown that these symptoms occur concurrently and influence patients’ physical functioning [48]. Patients with incurable oesophageal cancer have reported that their symptoms and concerns are interconnected in so far as pain and fatigue prevent them from participating in normal daily activities, and their functional limitations change their sense of identity and provoke a feeling of being a burden to others [49].

Previous research indicates that patients with incurable cancer can benefit from physical and psychosocial rehabilitation in terms of improved physical functioning, symptom control and increased QOL [50]. This indicates that, through palliative rehabilitation, it is possible to preserve patients’ physical functioning and thus relieve the burden of other symptoms and concerns. A programme for enhanced recovery is provided for patients with a curative disease, and a plan for pre-operative optimization is under development, but a strategy for maintaining physical functioning among patients with an incurable disease is lacking. This illustrates that these patients are managed in surgical care and that the current support system is structured and adapted for patients with a curative disease. However, it is important to provide proactive support also for those with an incurable disease.

In the present study the majority of patients reported severe anxiety among family and friends. This is in line with previous research showing significant levels of psychological distress among family and friends of patients with oesophageal cancer [51]. Research has also shown that anxiety is particularly intense at the time of diagnosis [52], especially if the prognosis is poor [53]. Our findings indicate that patients perceive that their family and friends are anxious early in the illness trajectory. As oesophageal and gastric cancer may progress rapidly, the patients’ family and friends have a very short time to adapt to the new life situation and lack of knowledge about patient care and difficulties to navigate the health care system and receive support are a source of considerable distress [54]. As patients´ and their families’ anxiety are significantly correlated [55], a family-centred approach embedded in palliative care could enhance the wellbeing of the whole family unit, once again highlighting the need to integrate a palliative care approach into standard surgical care.

This study has some strengths and several limitations that need to be addressed. A major strength is that data are based on validated and well-established instruments, the EORTC QLQ-C30 and QLQ-OG25. This ensures that the instruments measure what they are supposed to measure and it facilitates result comparisons with other studies. However, some limitations needs to be mentioned. Given the descriptive cross-sectional design, conclusions about causality/associations cannot be drawn and the results from the sub-groups analysis needs to be interpreted with caution as the small sample size may increase the risk for type II errors. The QOL comparison between the whole study population and the general population was not adjusted by sex and age. However, the published reference data that was used in the analysis was based on a sample that reflect the sex and age distribution of patients with oesophageal and gastric cancer [25, 36]. Another potential limitation is related to the representativeness of the sample in which 30.3% of the patients with oesophageal cancer and 33.3% of the patients with gastric cancer had a palliative treatment strategy. The fact that the proportion of patients receiving palliative treatment is lower in this study compared with register data [2] is likely to be related to the fact that some patients are so severely ill already at diagnosis so that they are not referred to the outpatient specialist clinic for diagnosis. This means that patients with more severe illness are probably underrepresented in this study and it is therefore likely that patients’ symptoms, problems and low QOL are underreported and underestimated. However, we appreciate that the sample is representative of patients diagnosed in outpatient care. Although eligible, some patients were not asked to participate. We experienced that the nurse at the outpatient department did not ask the patients to participate due to lack of time. However, this may not be a serious threat to the study’s validity as this was related to administrative factors, rather than patient-related factors.

## Conclusions

This cross-sectional study on patients newly diagnosed with oesophageal and gastric cancer found that patients are severely affected by their illness already at the time of diagnosis, in terms of reduced QOL and functioning, and several burdensome symptoms. Few differences were found between patients planned for a palliative and curative treatment strategy. The findings suggest that to help alleviate symptom burden and increase QOL for this group of patients an early palliative approach is needed for all, regardless of treatment intent.

## Data Availability

The datasets generated and/or analysed during the current study cannot be shared publicly due to regulations in the Swedish Data Protection Act (2018:218; 2019; 219) and Ethical Review Act (2003:460), but data are available from the corresponding author on reasonable request.

## Abbreviations

QOL: Quality of life
EORTC: European Organization for Research and Treatment of Cancer
IPOS: Integrated Patient Outcome Scale
ASCO: The American Society of Clinical Oncology
BMI: Body mass index
SD: Standard deviation
Q1: First quartile
Q3: Third quartile
ASA: American Society of Anesthesiologists
S-PCG: Swedish Palliative Care Guide
